# Removal of failed crown and bridge

**DOI:** 10.4317/jced.50690

**Published:** 2012-07-01

**Authors:** Ashu Sharma, G R. Rahul, Soorya T. Poduval, Karunakar Shetty

**Affiliations:** 1 BDS, MDS. Dept. of Prosthodontics. Bangalore Institute of Dental Sciences and Research Center. Bangalore, India.; 2 BDS, MDS. Professor and Head of Department of Prosthodontics. Bangalore Institute of Dental Sciences and Research Center. Bangalore, India.; 3 BDS, MDS. Professor, Department of Prosthodontics. Bangalore Institute of Dental Sciences and Research Center. Bangalore, India.

## Abstract

Crown and bridge have life span of many years but they fail for a number of reasons. Over the years, many devices have been designed to remove crowns and bridges from abutment teeth. While the removal of temporary crowns and bridges is usually very straightforward, the removal of a definitive cast crown with unknown cement is more challenging. Removal is often by destructive means. There are a number of circumstances, however, in which conservative disassembly would aid the practitioner in completing restorative/endodontic procedures. There are different mechanisms available to remove a failed crown or bridge. But there is no information published about the classification of available systems for crown and bridge removal. So it is logical to classify these systems into different groups which can help a clinician in choosing a particular type of system depending upon the clinical situation. The aim of this article is to provide a classification for various crown and bridge removal systems; describe how a number of systems work; and when and why they might be used. A PubMed search of English literature was conducted up to January 2010 using the terms: Crown and bridge removal, Crown and bridge disassembly, Crown and bridge failure. Additionally, the bibliographies of 3 previous reviews, their cross references as well as articles published in various journals like International Endodontic Journal, Journal of Endodontics and were manually searched.

** Key words:**Crown and bridge removal, Crown and bridge disassembly, Crown and bridge failure.

## Introduction

The use of crown and bridgework to restore a patient’s dentition is a treatment carried out by practitioners on a regular basis. Despite advances in the materials and technologies used to construct such restorations, and with the cements used to retain them, failure and the need to replace crowns and bridges occurs. The reasons for failure are multiple and caries are found to be most common cause. The longevity of prosthesis varies with type of prosthesis (1-6). Even the rough, over contoured crowns can lead to restoration failures (7). At times as the restoration gets damaged and attempts to repair them with different materials (8-10) and methods might fail. Such restorations needs to be removed.

In recent systematic review of the survival and complication rates of fixed partial dentures , the 10 year probability of survival was 89.1% (11).This finding was similar to two meta-analyses reported on in 1994 and 1998 (90% and 92%) (12-13).

Over the years, many devices have been designed to remove crowns and bridges from abutment teeth (14-19). These crowns and bridges may be fabricated from dental acrylics cemented to the abutment teeth with non-rigid temporary cements, or they may be definitive restorations fabricated from cast metal, porcelain-metal, ceramic, or composite resin cemented with more rigid cements. While the removal of temporary crowns and bridges is usually very straightforward, the removal of a definitive cast crown with unknown cement is more challenging. For a temporary crown or bridge, the restoration can be removed using a hand instrument, usually a scaler or large spoon excavator, or crown-removing pliers or a hemostat exerting force parallel to the long axis of the tooth. The crown or bridge is gently moved until the cement seal is broken. The restoration is then easily and atraumatically removed by breaking the weak cement seal between tooth and restoration.

## Search Strategy

A PubMed search of English literature was conducted up to January 2010 using the terms: Crown and bridge removal, Crown and bridge disassembly, Crown and bridge failure. Additionally, the bibliographies of 3 previous reviews, their cross references as well as articles published in International Endodontic Journal, General dentistry journal, Journal of Prosthodontics, Journal of Clinical Periodontology, British Dental Journal, Journal of Endodontics, Journal of prosthetic dentistry and Dental Update were manually searched.

## Crown and Bridge Failure

There are multiple causes for crown and bridge failure (20-21). These causes of crown and bridge failures can be classified into three main groups:

1.Biological. 2. Mechanical. 3. Aesthetical ( Table 1).

**Table 1:**
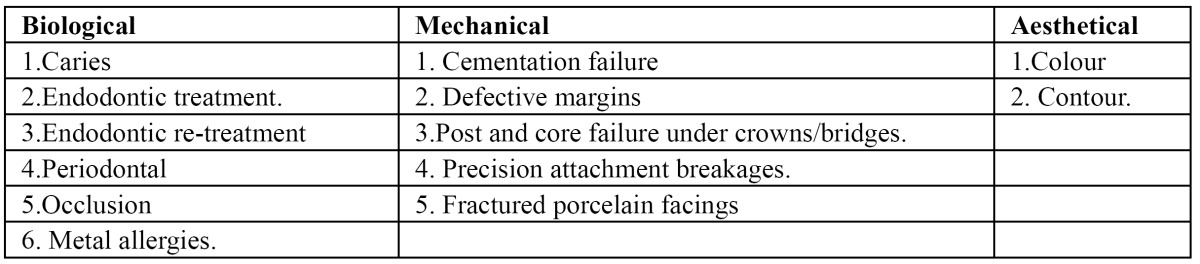
Classification of causes of crown and bridge failures.

## Clinical Considerations for Conservative Approach to Disassembly

The provision of crown and bridgework for patients can be time consuming and expensive. Whilst there are ties when teeth associated with crowns or bridges are beyond salvage, e.g. gross caries and severe periodontal bone loss, there are circumstances where a conservative approach to crown and bridge removal may aid the clinician and/or reduce the financial burden on the patient. These include:

a. Endodontics: Endodontic treatment or re-treatment completed with an access cavity cut through an extracoronal restoration may contribute to failure. Without its removal, a clinician cannot be absolutely certain of eliminating contributing pathological factors which may not be apparent from a clinical or radiographic examination. Even with the use of operating microscopes, endodontic access through a crown or bridge abutment is more difficult and destruction of unnecessary tooth structure is more likely. Further advantage include: better visualization of tooth morphology, ease of radiographic interpretation of the chamber and better visualization of fractures (15).

b. Failure of cementation of a retainer(s) on an otherwise sound bridge. Consideration should always be given to the reason for the failure before recementation. Whilst this is outside the remit of this article reasons include:

Inadequate tooth preparation.

Poor fit of the restoration.

Poor cementation.

Occlusal factors.

Differential mobility between abutments.

Inappropriate design of restoration.

Inappropriate choice of cementation material.

c. Decementation of one retainer of a resin-retained bridge where a fixed-fixed design is considered necessary. This may include post-orthodontic treatment in patients suffering from hypodontia or cleft palate.

d. The retrieval of cement-retained crown and bridge suprastructures on implants, following loosening of an abutment screw underneath the restoration. The incidence of this is low (4%) (22-24) but could be a potentially expensive complication if the supra structure could not be retrieved.

e. Decementation of resin-retained bridgework being utilized as a provisional restoration during the stages of providing a single tooth implant- retained crown.

f. Crowns and (to a lesser extent) bridges are occasionally designed with milled surfaces or intra- or extra-coronal precision attachments incorporated. Destructive removal of such structures can render the denture unusable and be costly and time consuming to replace. Conservative removal may allow them to be reused.

g. removal of temporary or provisional crowns and bridges is not always straightforward. Conservative removal may be beneficial to a treatment plan where their reuse is important.

h. Large span bridges on multiple retainers in which one or more are failing and require removal. Destruction of the entire prosthesis may make temporization difficult.

## Considerations before Deciding on a Crown Removing System

For deciding on a particular system, a careful assessment of the patient and the status of his/her teeth need to be made. One should consider the following things prior to crown and bridge removal ( Table 2).

**Table 2:**
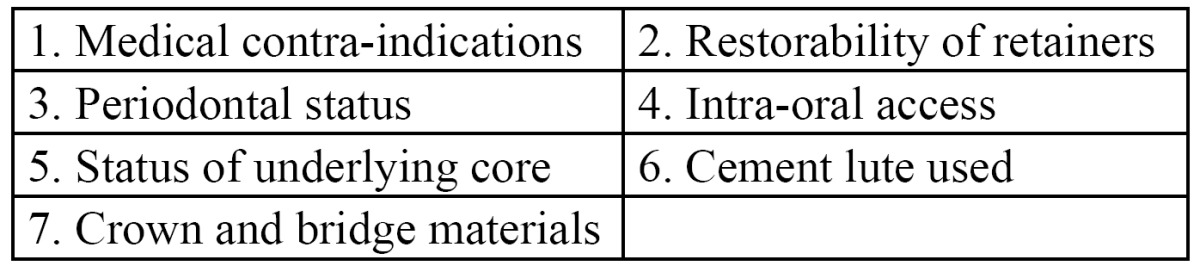
Factors considered prior to crown and bridge removal.

The use of ultrasonics is contraindicated in patients with hepatitis-B, herpes and cardiac pacemakers (25). Periodontal support and mobility is assessed before considering the use of a technique. The restorability of the tooth is also considered. The intra-oral accessibility is also considered because some techniques require adequate accessibility else there might be damage to the opposing dentition. Knowledge of the underlying core material is also very useful when considering applying traction forces. This, however, is not always possible as you may be removing another clinician’s work. Misdirected forces could damage the underlying tooth or core. Forces of removal should be applied along the path of withdrawal to reduce the risk of abutment fracture. A risk assessment between salvaging the restoration and risking damage to the supporting abutment needs to be done. Aesthetic failures such as fractured porcelain facings, could be managed more economically if the crown or bridge-work was retrievable, particularly if intra-oral attempts (26) of repair had been unsuccessful.

## Crown and Bridge Disassembly Classification

There are different mechanisms available to remove a failed crown or bridge. But there is no information published about the classification of available systems for crown and bridge removal. So it is logical to classify these systems into different groups which can help a clinician in choosing a particular type of system depending upon the clinical situation. The systems can be grouped into three categories:

1. Conservative: Prosthesis remains intact. It works in general by applying a percussion or traction force, breaking the luting cement and enabling the prosthesis to be removed.

2. Semi- conservative: Minor damage to the prosthesis is done but still it could potentially be reused. These techniques involve cutting a small hole in the prosthesis, enabling a force to be applied between the preparation and the bridge to break the luting cement.

3. Destructive: Prosthesis damaged and not reusable. The crowns is sectioned which enable sit to be levered off (1) ( Table 3).

**Table 3:**
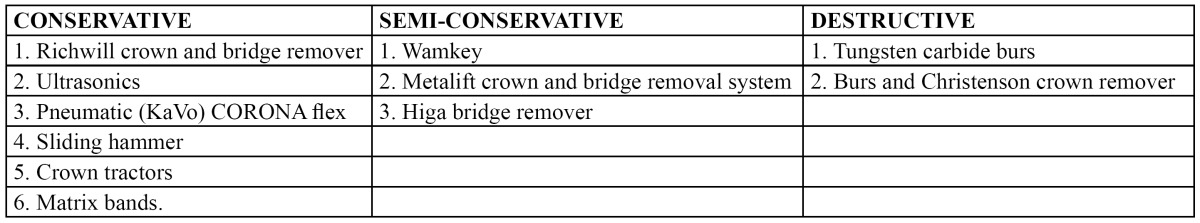
Classification of crown and bridge removal systems.

I. Conservative Disassembly

1. Richwill crown and bridge remover:

It is a thermoplastic resin that has been advocated for the removal of crowns and bridges (27).The resin is softened in hot water then placed interoclusally. Patient is asked to bit on it till the resin block gets compressed to two-thirds its bulk. This is then cooled with water with triple spray syringe until it is hard. Patient is now instructed to open mouth rapidly and forcefully. This technique has been reported to be 100% successful for temporary crowns (28) and 60% successful for the dislodgement of cast restorations in conjunction with the application of ultrasonic energy.

2.Ultrasonics:

The application of ultrasonic energy to remove cast restorations by disrupting the cement lute is based on its efficacy in removing metal posts (29). The application of ultrasonic energy alone, or in conjunction with other techniques, can be successful in removing restorations.

3.Pneumatic (KaVo) CORONAflex:

The technique for removing bridges using brass wire threaded through bridge embrasures to form a loop on to which a force can be applied to dislodge a bridge is not without its risks (18). These are similar to the use of the sliding hammer designed crown and bridge removers. Cores could be fractured and periodontally involved teeth could be extracted. The CORONA flex crown and bridge remover is a modification of this approach.

It is an air-driven device that connects to standard dental airline. It works by delivering a controlled low amplitude shock at its tip along the long axis of the abutment tooth. The loop is threaded under the connector and the tip of the crown remover is placed on the bar. The impact is activated by removing the index finger from the air valve on the hand piece. The kit also includes clamps that can be attached to individual crowns with autopoly-merizing resins; the impact is subsequently applied via the clamp to dislodge the crown (1).

**Figure 1:**
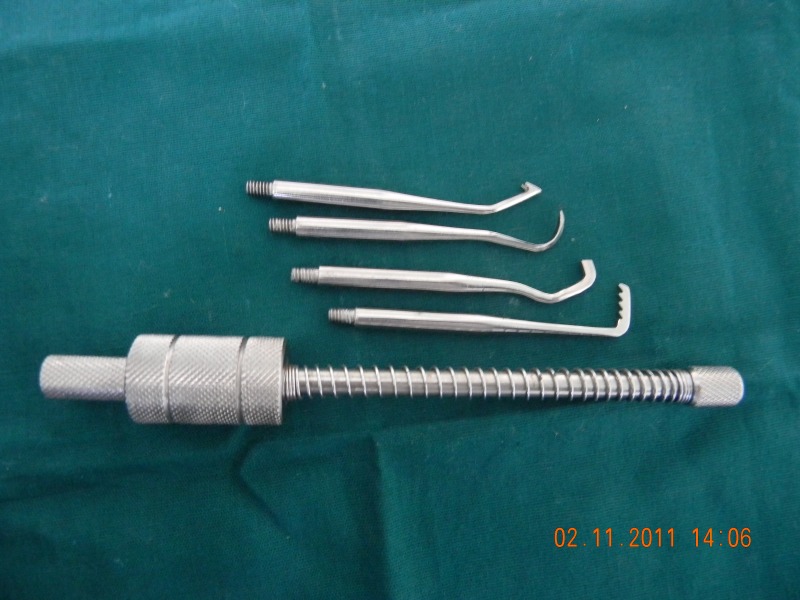
Sliding Hammer Type Crown Remover with different attaching tips.

**Figure 2:**
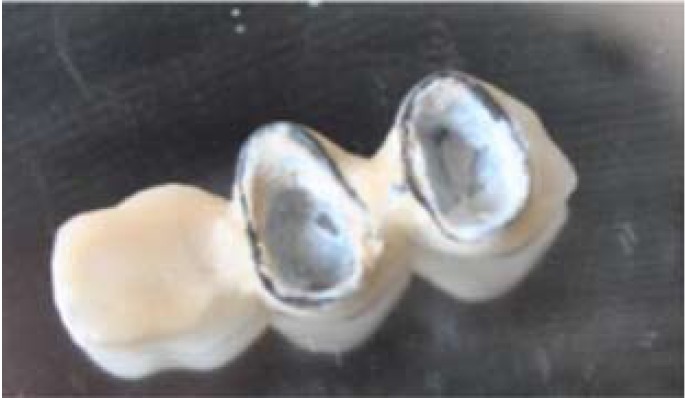
Conservatively removed cantilevered bridge which can be re-used again.

4. Sliding Hammer:

The basic principle of sliding hammer is that a suitable tip is selected to engage the crown margin and then a weight is slid along the shaft in a series of short, quick taps to loosen the restoration. Various sliding hammer designs are available in market. The use of this system can be uncomfortable for the patients and their use has been considered less reliable. This technique is not recommended for patients with periodontally involved teeth owing to the risk of unintended extraction. Damage to porcelain margins is also likely with such techniques (1) (Fig. 1, Fig. 2).

5. Crown Tractors:

Crown tractors grip the restoration with the aid of rubber grips and powder designed to dislodge the restoration without damaging the restoration. This is a particularly effective system for removing provisional crowns, crowns that have been cemented with temporary cement, or crowns that are difficult to remove at the try-in stage. The soft grip reduces the risk of damaging porcelain margins (1).

**Figure 3:**
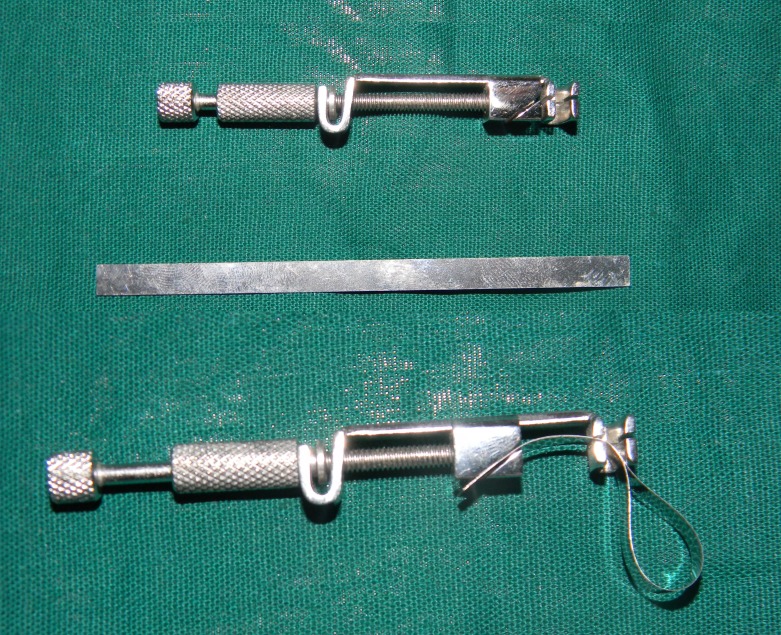
Siqveland Matrix Band.

6. Matrix Bands:

The application of a Siqveland Matrix Band over the crown, which is burnished into the undercuts and then pulled vertically, can be a successful technique for careful removal (30). (Fig. 3)

II. Semi-Conservative Disassembly

Attempts to remove restorations as mentioned above without damaging it may not be successful or the pulling device may be unpleasant experience for the patient. A semi-conservative approach is applied in such cases in which a small amount of damage is done to the restoration; the advantage is that this then allows a more controlled and less traumatic application of force to dislodge the casting.

1. Wamkeys:

Wamkeys are simple narrow-shanked cam devices available in three sizes. The clinician cuts a hole through the crown or retainer parallel to the occlusal surface and at the imagined level of the underlying core. A suitably sized wamkey is inserted with the broadest surface of the cam parallel to the occlusal surface, until it is centrally placed when it is rotated about the axis of the shank through 90 degrees. The force produced should be in the path of insertion of the crown or retainer which is easily dislodged. It is important not to attempt to lever off the crown with the instrument and it can be difficult to locate the interface between the occlusal surface of the core; the approach should be first to identify the cement layer before extending the channel across the occlusal surface. The restoration can be recemented and the hole filled with plastic filling material (1).

2. Metalift System:

This system is based on the “jackscrew” principle; a precision hole is drilled through the occlusal surface of a cast restoration, the area around the periphery of the hole is undermined before a threaded screw is wound into the space (1,28). A thread is cut in the metal of the casting and, when the instrument is stopped from advancing by contact with the underlying core, continued rotation of the screw results in a jacking force that displaces the crown from the preparation.

Metal ceramic prosthesis can be removed using this system, although care should be taken to remove enough ceramic from the area where the hole is to be drilled so as to minimize the risk of fracture. The minimum thickness of metal required is approximately 0.5mm the complete kit includes precision attachments to make good the hole prior to recementation. The damage is repaired with a plastic filling material.

III. Destructive Disassembly

Disassembly by means of cutting through the crown with a tungsten carbide diamond bur is probably common practice for most clinicians. Confining the slot to the labial surface, and applying an ultrasonic instrument to disrupt the cement lute, can provide space to elevate the crown and bridge so that it remains intact. Where adhesive cements are used it becomes necessary to section through the lingual surface as well, which will destroy the crown completely.

Whilst excavators and Mitchell’s Trimmers can be used, a useful instrument for this final stage is the Christen-son Crown Remover. The application of such a crown splitter spreads the split evenly, reducing the stress on the tooth/core (1).

## Conclusion

The article emphasized on general issues and concepts in crown and bridge disassembly, whilst at the same time focused on some specific devices and systems. Success lies in careful treatment planning; there will be situations where conservative approach is advantageous and situations where such attempts are contraindicated. None of the systems mentioned here are universally applicable. Therefore, it is important to adopt a flexible approach, that is, when you fail in removing crown and bridge by using one system then other systems should be tried. Patients should be made aware, at the outset of treatment, of the unpredictability of attempts at conservative and semi-conservative crown and bridge removal, and that there is always the possibility that a destructive approach is required. It is also very important to make risk-benefit analysis when considering conservative or semi-conservative disassembly and inform the patient of those risks.
